# Children’s physical activity during six weeks of school recess in hot weather: Associations with perceived and measured heat

**DOI:** 10.1371/journal.pgph.0007432

**Published:** 2026-11-11

**Authors:** Kylie Wilson, Jennifer Vanos, Chris C. Lim, Allison Poulos

**Affiliations:** 1 University of Arizona Cancer Center, Tucson, Arizona, United States of America; 2 School of Sustainability, College of Global Futures, Arizona State University, Tempe, Arizona, United States of America; 3 Mel and Enid Zuckerman College of Public Health, The University of Arizona, Tucson, Arizona, United States of America; 4 School of Technology for Public Health, Arizona State University, Phoenix, Arizona, United States of America; 5 College of Health Solutions, Arizona State University, Phoenix, Arizona, United States of America; Aga Khan University Medical College Pakistan, PAKISTAN

## Abstract

Regular physical activity supports children’s health, yet extreme heat threatens outdoor movement opportunities during school, particularly in regions prone to hot weather. We examined fourth- and fifth-grade students’ self-reported physical activity and associations with perceived and objectively measured heat during outdoor recess across the first six weeks of the 2024–2025 school year in five elementary schools in the Phoenix Metropolitan Area (Arizona, USA), where temperatures frequently exceed 37°C. Students (n = 170) completed 981 brief paper questionnaires twice weekly post-recess, reporting activity intensity (sedentary, light, moderate-to-vigorous) and perceived heat (“not at all,” “a little,” “a lot”); air temperature data were collected from nearby weather stations. On average, 35% of students spent most of recess sedentary, 43% in light activity, and 23% in moderate-to-vigorous activity, with 45% of responses rated as the highest perceived heat category. Cumulative link mixed models showed activity levels did not change significantly over time. In perceived heat models, higher perceived heat was associated with increased odds of higher activity (*β* = 0.89, SE = 0.34, *p* = 0.009); students who reported feeling “a little” or “a lot” hot were more active than those who reported being “not at all” hot. In objective heat models, higher temperatures were similarly associated with greater activity (*β* = 0.20, SE = 0.10, *p* = 0.045). For each 1°C increase, the odds of reporting the next higher perceived heat category increased 8% (OR=1.078, 95% CI:1.01-1.15). Contrary to expectation, children did not reduce activity intensity in relation to heat; higher perceived and measured heat were instead associated with greater activity, highlighting a potential mismatch between behavior and heat-related risk. Proactive strategies like shaded areas, schedule adjustments, adequate hydration, and heat adaptation plans should be considered to safeguard children’s health. Future research should include device-based activity and physiological measures, longer observation periods, causal study designs, and additional youth age groups or thermal conditions to inform best practices.

## Introduction

Regular physical activity protects against acute and chronic non-communicable diseases and reduces all-cause morbidity and mortality [1,2]. Among youth, active lifestyles are associated with improved cardiometabolic and mental health, healthy weight trajectories, and cognitive functioning [3–6]; yet fewer than one-quarter of school-aged youth in the United States of America (USA) meet recommended aerobic activity guidelines [7,8]. Because children and adolescents spend much of their waking hours at school, these settings are salient for promoting daily movement. Schools provide routine opportunities for physical activity, particularly through outdoor recess, which is often the primary source of unstructured physical activity during the school day [9]. However, access to safe and consistent outdoor activity is shaped by environmental conditions, and extreme heat increasingly threatens these opportunities [10], especially in regions prone to prolonged periods of hot weather.

Younger children may be uniquely vulnerable to heat-related illness during high temperatures due to physiological, behavioral, and developmental factors [11], and schools often lack sufficient resources or guidance to support outdoor movement during extreme weather [12]. Young children (<10 years old) differ physiologically from adults in ways that affect thermoregulation, such as lower sweating rates, a greater proportion of cardiac output directed to the skin, and greater heat production per unit body mass occurring during movement [13]. Evidence suggests that young children use different, rather than inherently less effective, thermoregulatory strategies; still, their vulnerability increases under more extreme environmental conditions [13]. Elevated heat loads (i.e., high ambient temperatures, radiant load, low wind, and/or high humidity) can increase physiological strain and risk of heat-related illness during outdoor exposure, complicating efforts to maintain safe opportunities for physical activity. However, the degree to which brief outdoor activity bouts (e.g., the 15–30 minute recess periods common in elementary schools [14,15]), confer meaningful heat-related risk under hot, dry conditions is not well established; with appropriate supports, most healthy children can safely participate in outdoor physical activity across a wide range of hot conditions, provided hydration and recovery are adequately maintained [16]. Prior research indicates children’s physical activity during recess declines markedly at temperatures above 32.8°C (91°F; [17]), yet in the desert Southwest (USA), outdoor recess frequently continues at temperatures exceeding 37.8°C (100°F, though generally low humidity) with children spending comparable time engaged in moderate-to-vigorous physical activity compared with more temperate conditions (e.g., 30°C/86°F; [15]). Recent systematic observation data from five elementary schools in the Phoenix metropolitan area (AZ, USA) showed that on high-heat days (33–43°C / 91–109°F), children remained moderately active during outdoor recess, spending about 28% of recess time in moderate-to-vigorous physical activity. Both girls and boys engaged in meaningful physical activity despite extreme heat, suggesting that children’s activity may persist under hot conditions [18].

At the same time, overexposure to heat can pose health risks, particularly when paired with activity, and school personnel must often make decisions about recess with limited support and guidance. This issue is particularly concerning as outdoor activities often continue during periods of high heat; for example, recent observational data from elementary schools in the desert Southwest (AZ, USA) showed that on days ranging from 33-43°C (91–109 °F), 60% of observed recess periods were held outdoors [18]. Even in states with policies mandating daily recess [19], limited access to shade structures or vegetation, flexibility to shift recess timing or move activities indoors during peak heat hours, and adequate alternative indoor spaces (e.g., multipurpose cafeteria-gymnasium spaces) complicate efforts to ensure recess provides safe opportunities for meaningful physical activity. Results from a study of schools in Maricopa County (AZ, USA) suggest that heat-related recess decisions can vary widely, even among schools within the same district, reflecting limited consistency in how schools respond to high temperatures [10]. As extreme heat becomes more frequent and intense, public health agencies have begun developing structured guidance for schools. For example, the Arizona Department of Health Services (ADHS) has issued heat guidance grounded in a Hierarchy of Controls framework that prioritizes system-level protections such as limiting outdoor exposure and modifying schedules over individual behaviors, while also identifying administrative controls such as scheduled rest and hydration breaks as key supports [20] - as poor hydration status is among the primary modifiable risk factors for exertional heat illness in children [16]; California has similarly developed a statewide Extreme Heat Action Plan for schools [21].

Concerns about heat exposure in schools are ongoing, yet few studies have examined children’s activity patterns across repeated extreme heat events or chronically hot climates, how children’s perceptions of heat are associated with their behavior, or how perceived heat aligns with measured air temperature. Understanding these dynamics is essential for moving toward evidence-informed school-based heat adaptation strategies that protect children’s health while preserving opportunities for physical activity. The purpose of this study was to (1) examine children’s self-reported physical activity during outdoor recess across the first six weeks of the school year (August-September 2024) in extreme heat, (2) evaluate whether children’s perceptions of heat or measured air temperature moderated changes in activity over time, and (3) assess whether children’s perceptions of heat corresponded with measured air temperatures.

## Materials and methods

### Study design

This study used a longitudinal design to study changes in children’s self-reported physical activity intensities and heat perceptions during outdoor school recess. Data were collected during the first six weeks of the 2024–2025 school year (August-September) from children at five elementary schools located in the Phoenix Metropolitan Region (AZ, USA), an area characterized by persistently high late-summer and early-fall temperatures. During this period, ambient temperatures frequently exceeded 37.8°C (100°F). The study coincided with an exceptional heat season in Phoenix, which experienced both its hottest September and hottest year at the time (2024), as well as the longest consecutive run of days with maximum temperatures at or above 37.8°C (100°F) documented to date [22,23].

Decisions regarding whether recess was held indoors or outdoors were made independently by each school’s administration, resulting in variability in recess location across days. Only surveys completed after outdoor recess sessions were included in this study.

### Participants and setting

Schools were randomly selected from a pre-specified sample of 57 schools in the Phoenix Metropolitan Area that had previously completed an external survey indicating that extreme heat affected outdoor recess practices [10]. The initial pool was stratified by geographic location, either within the City of Phoenix or in the surrounding metropolitan area, to reflect the city’s representation of approximately 40% of the regional population. A random number generator in Microsoft Excel was then used to select schools from each stratum.

Principals at selected schools were invited to participate until five agreed to enroll in the study; seven schools declined participation. Eligible participants were students enrolled in fourth or fifth grade at each participating school. Classroom teachers at participating schools received a $25 gift card as compensation for administering student surveys, and each school was offered a $500 recess equipment bundle as an additional participation incentive. This incentive was provided uniformly to each school following participation in the study and was not contingent on student enrollment, response rates, or activity levels, minimizing the likelihood that it introduced participation or activity-related bias.

### Ethics statement

Principals at participating schools provided written authorization for study activities and distributed opt-out consent forms to parents and guardians of all eligible students; no opt-outs were received. Following written assent, students completed a brief paper-based questionnaire immediately after recess on Tuesdays and Thursdays for six weeks, from 1 August through 11 September 2024. The study protocol was approved by Arizona State University’s Institutional Review Board (STUDY 00020443).

### Measures

#### Reported physical activity.

Immediately following outdoor lunch recess, students self-reported the intensity of their physical activity using a three-point ordinal scale: sedentary (not breathing hard at all), light physical activity (breathing hard a little), and moderate-to-vigorous physical activity (breathing hard a lot), adapted from single item measure [24], reflecting perceived exertion*.*

#### Reported heat perceptions and measured air temperature.

Students also reported their perceived heat levels using a three-point scale on whether they felt “not at all hot,” “a little hot,” or “a lot hot” immediately after recess. This item was developed by the research team and was not formally pilot-tested; the item used the same three-point response format as an existing readiness-to-learn measure as part of a larger study addressing heat-health impacts to children [25]. Daily air temperature data corresponding to scheduled recess times at the participating schools were obtained from the nearest weather station using MesoWest (https://mesowest.utah.edu). Temperature values were treated as continuous measures in analyses.

### Statistical analysis

Analyses were restricted to students with data from at least two distinct measurement days to support longitudinal modeling. Of the original 1,006 surveys representing 193 students, 985 surveys from 172 students met this inclusion criterion. Two additional students (four surveys) were excluded because they were the only participants with repeated observations at one school, likely reflecting inconsistencies in survey administration at that site. As a result, data from four schools were included in the final analyses.

Descriptive statistics were used to summarize survey characteristics, temperature conditions, and distributions of physical activity intensity and perceived heat for all students. Changes in self-reported physical activity intensity over time were examined using cumulative link mixed models (CLMM) with a logit link and random intercept for student to account for repeated measures. One model evaluated the potential moderating effect of perceived heat by including an interaction between day and perceived heat, and a separate model examined the same interaction using measured air temperature.

Additionally, an ordinal logistic regression was used to examine the association between measured air temperature and students’ perceived heat, evaluating whether subjective perceptions corresponded with measured conditions. Given the negligible school-level clustering observed in the activity models (ICC < 0.5%), and that temperature varied primarily within- rather than between-schools during the study period, an ordinal logistic regression without school random effects was used for this model. Pairwise comparisons of perceived heat categories were adjusted for multiple comparisons using the Bonferroni method. Analyses were conducted in R (version 4.5.2; [26]) using the dplyr, tidyr, ordinal, MASS, emmeans, broom, ggplot2, and knitr packages [26–34]. Statistical significance was assessed at *p* < 0.05.

## Results

### Descriptive statistics

A total of 981 surveys were collected from 170 students, with individual participation ranging from 2 to 11 surveys per student (mean = 5.8 surveys). Across the four included schools, participation varied from 12 to 73 students per school and 45–490 surveys, with a mean of 3.5 - 8.6 surveys per student, depending on school. Outdoor air temperatures during recess across the study period for schools in our sample ranged from 32.2°C (90°F) to 42.2°C (108°F); mean = 36.6°C (97.9°F).

Recess duration ranged from 15 to 30 minutes across participating schools, and recess consisted of unstructured free-play time at all sites. Across the four schools included in analyses, average outdoor shade canopy coverage ranged from 13.5% to 53.9%. Outdoor recess areas totaled 3,079–18,932 square yards, with surfaces predominantly concrete (56% across all schoolyard areas) and grass (31%), with smaller proportions of dirt, wood chips, sand, and rubber. Across schools, students reported that an average of 34.5% of recesses were predominantly spent sedentary, 42.5% in light physical activity, and 23.1% in moderate-to-vigorous physical activity (MVPA). These distributions remained relatively consistent across the six-week period. Students also reported feeling “a lot hot” in 45% of observations. Descriptive statistics are shown in Table 1.

**Table 1:** Descriptive statistics of physical activity levels and perceived heat.

Variable	Item	N	Mean (% student-averaged)	SD (%)
Physical Activity	Sedentary	327	34.5	32.2
	Light	403	42.5	29.8
	Moderate-to-vigorous	219	23.1	28.8
Perceived Heat	Not at all	195	20.6	29.3
	A little	307	32.4	28.9
	A lot	445	47.0	35.2

*Note*. Missing observations: Physical Activity = 32 (3.3%), Perceived Heat = 34 (3.5%).

### Physical activity levels and associations with heat

Most variability in reported physical activity intensity occurred between students, with less than 0.5% of total variance attributable to schools, indicating minimal school-level clustering.

#### Perceived heat model.

Activity levels did not change significantly over time (Day: *β* = 0.014, SE = 0.014, *p* = 0.30; Fig 1). Higher perceived heat was associated with greater physical activity intensity (Perceived heat - linear: *β* = 0.89, SE = 0.34, *p* = 0.009) while quadratic effects and interactions with time were not significant, indicating that perceived heat did not meaningfully alter activity trajectories over the study period.

**Fig 1:**
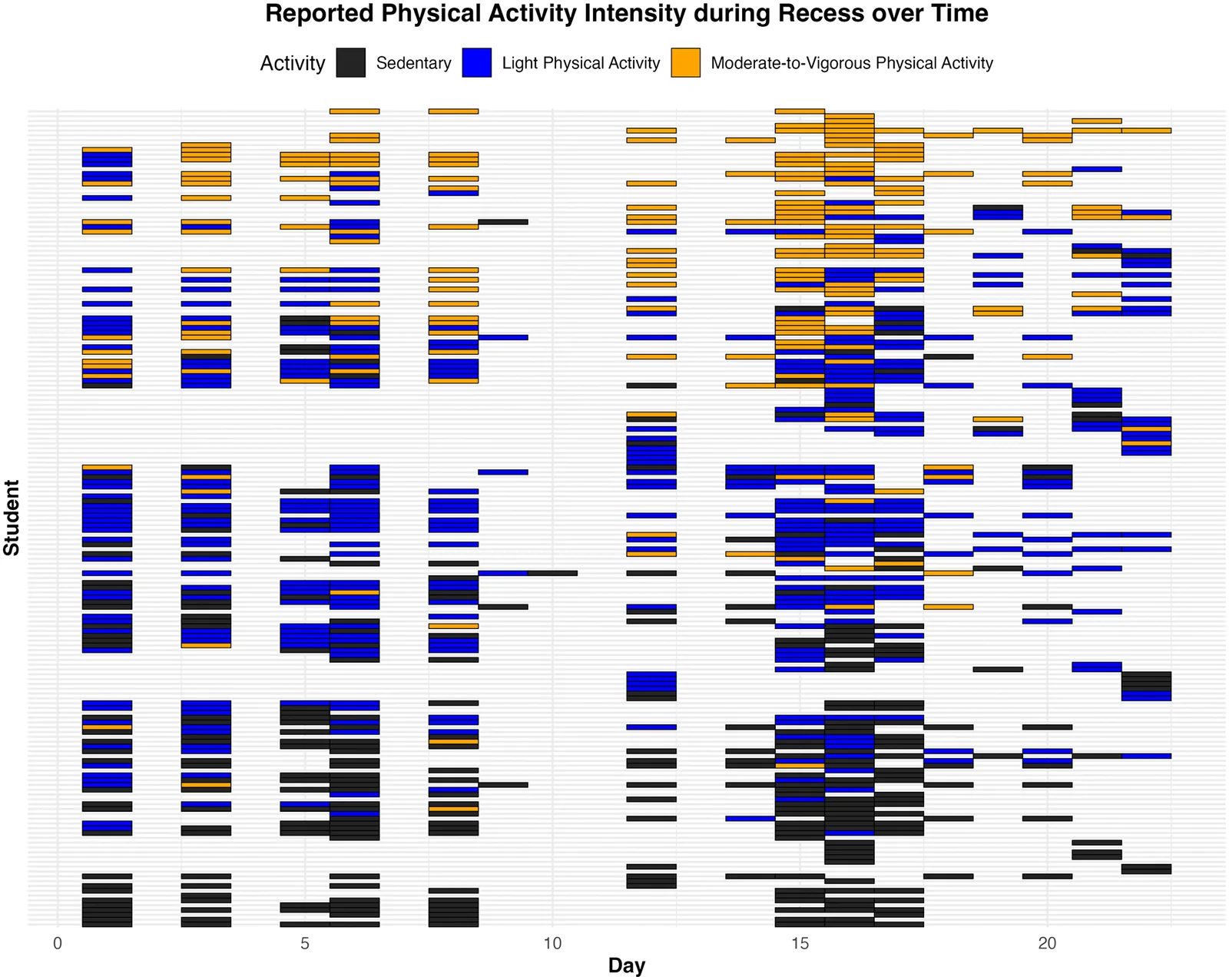
Reported physical activity intensity during recess across students over the study period.

Pairwise comparisons of perceived heat categories indicated that students reporting moderate heat (“A little”) were significantly more active than those reporting the lowest perceived heat (“Not at all”; *β* = -0.772, SE = 0.260, *z* = -2.973, *p* = 0.008). Students reporting the highest perceived heat (“A lot”) were significantly more active than both lower heat groups (vs. “Not at all”: *β* = -1.443, SE = 0.264, *z* = -5.475, *p* < 0.0001; vs. “A little”: *β* = -0.671, SE = 0.195, *z* = -3.440, *p* = 0.002). These differences were consistent across the six-week period, generally reflecting stability in the trajectories observed over time. These comparisons were averaged across the study period, as interactions between days and perceived heat were not significant.

#### Measured temperature model.

In a separate CLMM with measured (objective) heat, based on air temperature, higher temperatures were positively associated with greater activity intensity (*β* = 0.20, SE = 0.10, *p* = 0.045), such that each 1°C increase corresponded to a 22% increase in the odds of reporting a higher activity category (OR = 1.22, 95% CI: 1.01 - 1.49). To further illustrate this pattern descriptively, we examined the distribution of physical activity intensity (% sedentary, light, and moderate-to-vigorous) across three temperature bins (<35°C, 35–38°C, and >38°C). The proportion of surveys reporting moderate-to-vigorous physical activity increased across bins (12.5%, 21.2%, and 36.4%, respectively), while the proportion of light activity decreased (65.6%, 42.8%, and 34.8%) - consistent with the positive association observed in the regression model (Table 2).

**Table 2:** Physical activity intensity by temperature category during outdoor recess.

Temperature Category	N (surveys)	% Sedentary	% Light	% Moderate-to-Vigorous
<35°C (<95°F)	32	21.9	65.6	12.5
35–38°C (95–100°F)	778	36.00	42.8	21.2
>38°C (>100°F)	132	28.8	34.8	36.4

As in the perceived heat model, activity did not change significantly over time (*p* = 0.58), and the interaction between day and temperature was not significant (*p* = 0.61), indicating stable activity trajectories across repeated exposure to extreme heat.

### Alignment of perceived heat with air temperature

The results of the ordinal logistic regression showed measured air temperature was positively associated with students feeling hot (*β* = 0.072, SE = 0.035, *t* = 2.05, *p* = 0.040; Fig 2), such that each 1°C increase in temperature increased the odds of reporting a higher perceived heat category by approximately 8% (OR = 1.078, 95% CI: 1.01 - 1.15). The thresholds separating categories were 1.37 (Not at all | A little) and 2.89 (A little | A lot).

**Fig 2:**
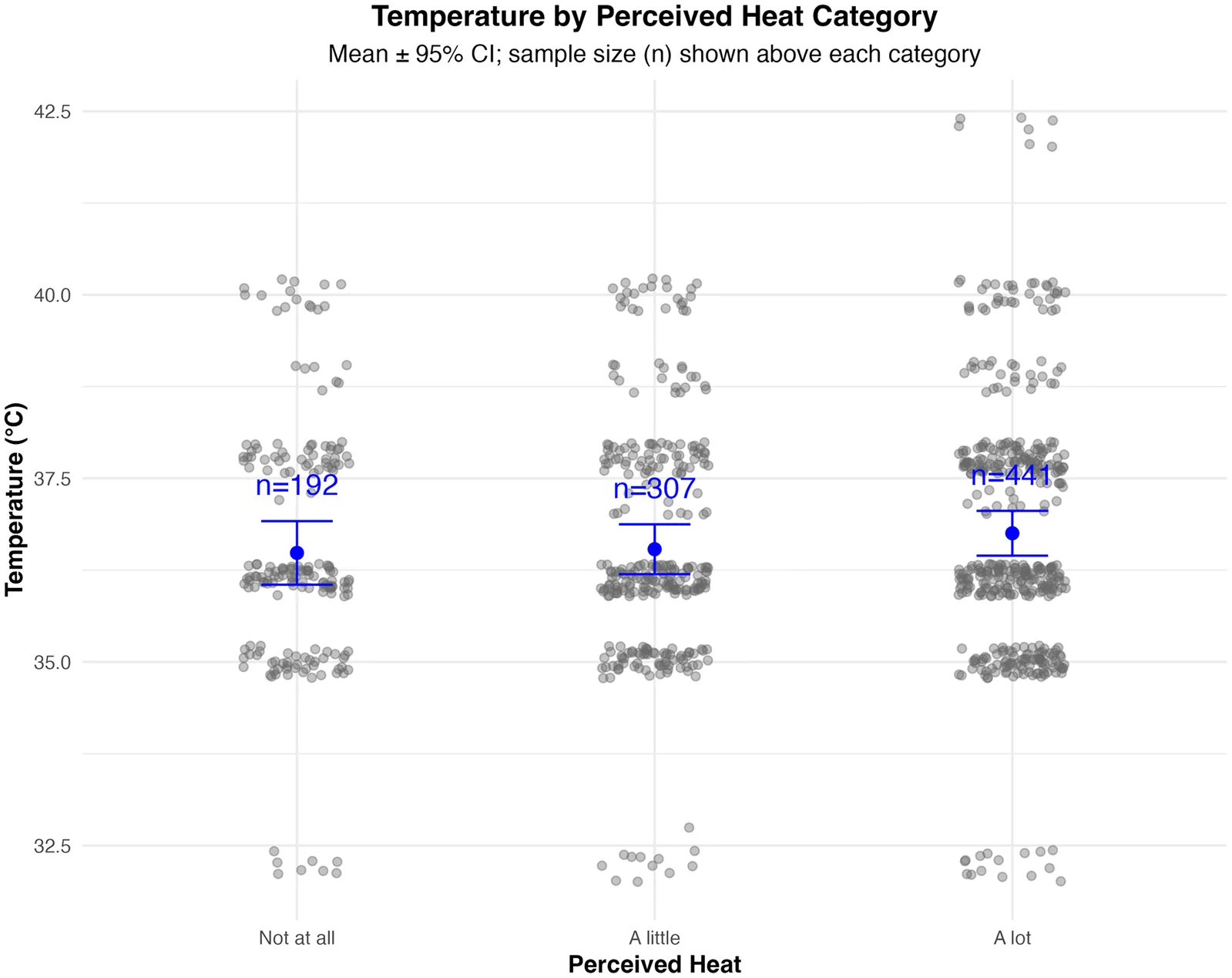
Relationship between outdoor temperature and children’s perceived heat during recess.

## Discussion

Across the six-week study period, students’ self-reported physical activity distributions remained stable, with roughly one-quarter of survey responses (23.1%) indicating that most of recess was spent in moderate-to-vigorous physical activity. Although outdoor temperatures were consistently high (32–42°C), perceived heat during recess did not appear to negatively affect activity intensity, and students’ reports of heat generally aligned with measured air temperatures, supporting perceived heat as a useful indicator of thermal conditions. Contrary to prior work [17], these findings suggest children may not necessarily self-regulate activity in response to extreme heat, highlighting a need for adult-led behavioral support and environmental interventions to ensure safe outdoor recess.

Prior research has consistently shown that children’s physical activity is associated with temperature in a curvilinear pattern, with activity peaking around 20–22°C (68–72°F) and declining at higher temperatures, particularly during unstructured or discretionary play [35–37]. School-based studies have further suggested that warmer conditions are associated with reduced moderate-to-vigorous physical activity during recess, alongside increased shade-seeking behavior, especially under extreme heat conditions; for example, in an Australian sample (max ~ 28°C/83°F in summer vs. ~ 15°C/59°F in winter), moderate-to-vigorous physical activity was 7.8 minutes lower per day on average in summer compared to winter [37], and in a Texas study, moderate-to-vigorous physical activity declined and shade-seeking increased markedly above 33°C (91°F) with effects steepening at higher temperatures [17]. Notably, the temperature-dampening effect on activity appears strongest in younger children [35], though the samples in the studies above are broadly comparable in age to our current study (~9–11 years), supporting the relevance of these comparisons

Contrastingly, however, our findings suggest that children in the desert Southwest may maintain meaningful levels of physical activity even under high-heat conditions. Despite average temperatures of 37°C, children remained active, spending 23.1% of recess in moderate-to-vigorous physical activity, comparable to levels reported in studies conducted in more temperate conditions (average ~30°C/86°F) using direct observation and accelerometry [15] and in a prior study of the same sample under similar high-heat conditions (33–43°C / 91–109°F) using systematic direct observation [18]. Moreover, perceived heat did not moderate activity trajectories across repeated exposures, with children who reported feeling hot engaged in more intense movement, as also found in the same age group in a Texas study [38]. These findings suggest that children aged ~9–11 years, even on hot days during school recess, may have a high motivation to be active and do not necessarily reduce their physical activity in response to heat. Maintaining activity under these conditions, even when children report feeling hot, may increase the risk of heat-related illness in the absence of external safeguards (e.g., environmental modifications, schedule changes, behavioral supports). However, further research is needed to assess whether a short bout (~15–30 minutes) of physical activity in high heat is detrimental, given the physical activity and acclimatization benefits, particularly if activity occurs in the shade and if hydration is maintained.

Several mechanisms may help explain these patterns of increased physical activity with actual and perceived heat. Children may exhibit a strong intrinsic motivation to play during recess after being sedentary for an extended period in often cool indoor environments that outweighs their thermal discomfort, particularly in school settings where recess time is limited and highly valued. At the same time, younger children in the 9–11 years age range may have a limited capacity to accurately interpret or act upon heat-related risk, even when subjective discomfort is present.

In our study, air temperatures during recess were consistently high, ranging from 32.2°C - 42.2°C (90.0°F - 108.0°F), resulting in limited environmental variability across observation days. This limited variability in air temperature likely contributed to the observed relationship between measured temperature and perceived heat. Because recess exposure was brief (~15–30 minutes), the duration may have been insufficient to meaningfully elevate heat-related illness risk, which may help explain why activity levels remained stable.

Importantly, the positive association between perceived heat and activity does not indicate causal directionality. Because perceived heat was reported after recess, children’s responses likely reflect their thermal experience following physical exertion rather than heat directly shaping activity decisions. Thus, it is therefore possible that children who were more active experienced greater physiological heat load and subsequently reported higher perceived heat, rather than perceived heat dictating activity levels. This interpretation is consistent with experimental and observational evidence demonstrating that exertion amplifies thermal sensation through increased metabolic heat production and cardiovascular strain [38,39]. Given that data collection in our study occurred at the start of the school year (mid-summer) and children were only exposed to brief, intermittent periods of outdoor activity during recess, partial heat acclimatization may have occurred, but full physiological acclimatization to the observed temperatures from recess alone is unlikely [40].

Overall, our findings highlight a disconnect between children’s activity behavior and environmental heat risk during outdoor recess. Children continued to engage in more vigorous physical activity under extreme heat, even when they reported feeling hot, and did not change activity intensity across repeated heat exposures. Because children in school settings have limited capacity to independently regulate their thermal environment and may not have full access to critical protective resources (e.g., shade, water), these findings underscore the importance of proactive, adult-guided strategies to support heat-adaptive physical activity in schools rather than reliance on children’s self-regulation. Emerging frameworks such as the ADHS Hierarchy of Controls [20], California’s Extreme Heat Action Plan [21], and HeatReady™ Schools [41] offer structured approaches to balancing continued physical activity with heat safety by prioritizing system-level protections before relying on individual-level behaviors.

### Limitations

This study has several limitations. First, the observational design precludes causal inference. Because perceived heat was reported immediately after recess, it may reflect children’s physical exertion rather than heat directly influencing activity levels. Second, data collection occurred during an exceptional heat season, with air temperatures consistently high and narrowly distributed (32.2-42.2°C); no comparison data from milder weather for these schools were collected. As shown in Table 2, a small proportion of surveys (n = 32) occurred below 35°C, limiting our ability to characterize activity patterns across a broader range of temperatures. Third, relative humidity and other environmental contributors to thermal strain (e.g., wind, solar radiation, shade or water access and use) that may influence children’s thermal experience and related behaviors were not measured and should be considered in future research. Of particular consideration is that hydration status was not measured in this study; most children do not meet recommended water intake under typical conditions, and physical activity and heat strain further increase fluid needs in ways that have not been well studied in children [42]. Given that poor hydration is among the primary modifiable risk factors for exertional heat illness [16], future studies should assess children’s fluid intake and water access in the schoolyard alongside activity and heat exposure measures. Fourth, school-level differences in recess duration, shade coverage, and surface composition were characterized descriptively (Table 1) but not included as covariates in the activity models; because schools with more shade or shorter recess may be more likely to hold outdoor recess on the hottest days, this could confound or attenuate the observed heat-activity association. Fifth, our mixed-effects modeling approach accounted for stable between-student differences in overall activity level via a student-level random intercept but did not directly assess preexisting activity disposition (e.g., general enjoyment of outdoor play), which may further explain individual variation in activity across temperature conditions. Finally, this study was conducted in the Phoenix metropolitan area (AZ, USA), a hot, arid desert climate; findings may not generalize to more humid climates, where the same air temperature can produce different physiological heat strain and perceived heat responses.

## Conclusions

Children’s physical activity during recess remained relatively consistent over repeated exposures to extreme heat. While perceived and measured heat were positively associated with activity, activity trajectories did not change meaningfully over time. These findings suggest that, even when children report feeling hot, they may continue to engage in physical activity highlighting a potential mismatch between activity patterns and heat-related health risks, as well as the role of adult-guided protections in hot school environments. It is important to note, however, that because perceived heat was reported immediately after recess, this observed association may partly reflect children’s physical exertion elevating their sense of heat, rather than heat shaping how active they were. As such, these findings should be interpreted as associations rather than evidence of a causal pathway from heat to activity.

Future research should incorporate device-based measures of physical activity and physiological strain, such as accelerometry, heart rate monitoring, and core temperature assessment, alongside self-report contextualization and additional environmental metrics (e.g., shade cover, humidity, and surface temperature) to provide a more comprehensive understanding of children’s activity and heat exposure experiences. Future studies should also assess children’s baseline activity preferences and disposition toward outdoor play, which could help clarify whether individual differences in activity levels are driven by heat exposure or by pre-existing behavioral tendencies. Longer observation periods, causal study designs, and consideration of biological or training/acclimatization adaptations may help elucidate activity patterns under extreme heat. Researchers should also consider examining other youth age groups in similar settings, as heat sensitivity and activity responses may differ.

From a practical standpoint, our findings suggest that children do not necessarily reduce their physical activity as heat increases, meaning schools cannot assume students will self-regulate their exposure during outdoor recess. Rather than pointing to a single temperature threshold at which recess should be modified, our findings support to the broader need for more comprehensive evidence before more firm, generalizable guidance can be established. In the interim, existing frameworks such as the ADHS Hierarchy of Controls, California’s Extreme Heat Action Plan, and HeatReady™ Schools offer structured, adult-driven approaches (e.g., shade provision, hydration access, schedule flexibility) that do not depend on children’s own heat-regulation behavior.
